# Dataset on bitcoin carbon footprint and energy consumption

**DOI:** 10.1016/j.dib.2022.108252

**Published:** 2022-05-11

**Authors:** Samuel Asumadu Sarkodie, Phebe Asantewaa Owusu

**Affiliations:** Nord University Business School (HHN). Post Box 1490, 8049 Bodø, Norway

**Keywords:** Bitcoin energy consumption, Bitcoin carbon footprint, Bitcoin econometrics, Cryptocurrency, Blockchain, Bitcoin carbon emissions

## Abstract

Due to data limitations on bitcoin-related emissions, assessing the environmental impacts of bitcoin appear difficult. This data in brief article presents constructed daily frequency dataset on bitcoin annualised carbon footprint spanning July 7, 2010 to December 4, 2021 with 4,158 observations. The 12 data variables capture floor, ceiling, and optimal annualised carbon footprint from coal, oil, gas, and the average from the 3 sources. The constructed bitcoin carbon footprint data are measured in kgCO_2_ using emission factors for electricity generation from IEA World Energy Outlook. The data will benefit multidisciplinary research on cryptocurrency from environmental, energy, and economics disciplines.

## Specifications Table

**Table:** 

Subject	Economics, Econometrics and Finance
Specific subject area	Cryptocurrency and Fintech
Type of data	Tables, and Figures
How the data were acquired	Bitcoin energy consumption data were acquired from the Cambridge Centre for Alternative Finance. The raw data is processed and modelled to produce bitcoin carbon footprint using STATA (version 16) and R (version 4.1.2) software.
Data format	Raw and analysed data formats submitted alongside the data article
Description of data collection	The daily frequency data capture 4,158 observations from July 7, 2010 to December 4, 2021. First, the raw data has 3 variables namely minimum, maximum, and optimal bitcoin annualised energy consumption. The energy consumption variables capture the total annual electricity consumption of the Bitcoin proof-of-work consensus network expressed in kilowatt-hours (kWh). The annualised measure of electricity assumes continuous power usage (i.e., minimal, maximal, and optimal) over one year period––with a subsequent application of a 7-day moving average to control for short-term hash-rate variabilities (Ref: CBECI, 2021). Second, 12 daily frequency data variables of bitcoin carbon footprint are constructed (see details in materials and method) using the 3 bitcoin annualised energy consumption data. Four different assumptions are used to generate the new variables. We assume energy used for data centres and electricity for mining equipment are derived solely from (1) coal, (2) oil, (3) gas, and (4) mixture/average from the 3 sources. The constructed bitcoin carbon footprint data are measured in kgCO_2_.
Data source location	• Institution: The Cambridge Centre for Alternative Finance (CCAF), University of Cambridge Judge Business School • City/Town/Region: 10 Trumpington Street, Cambridge • Country: UK
Data accessibility	*With the article* Data are available in Microsoft Excel Workbook format (.xlsx) for raw and analysed variables attached as supplementary material. Repository name: Figshare Direct URL to data: https://doi.org/10.6084/m9.figshare.19442933.v1

## Value of the Data

• The dataset consists of daily frequency measurements on bitcoin annualised carbon footprint with huge data points spanning July 7, 2010 to December 4, 2021.
• The dataset can facilitate empirical research on environmental and energy sustainability of bitcoin, thus, improving the global debate.
• The data can benefit multidisciplinary research on cryptocurrency from environmental, energy, and economics disciplines.
• The estimation of bitcoin carbon footprint using global parameters makes it generally applicable and reusable in any crypto-based studies on bitcoin sustainability assessment.

## 1 Data Description

Table 1 presents the data description of the 12 data variables constructed using 3 initial raw data from CBECI [1]. The dataset comprises daily frequency variables with their units of measurement.

**Table 1:** Sampled data description.

Abbrev	Variable description	Unit	Source
BTCENEMAX	annualised BTC electricity consumption (maximum)	kWh	CBECI [1]
BTCENEMIN	annualised BTC electricity consumption (minimum)	kWh	CBECI [1]
BTCENEGUE	annualised BTC electricity consumption (optimal)	kWh	CBECI [1]
BTCEMI_MAX	annualised BTC average emissions (maximum)	kgCO_2_	Authors
BTCEMI_MIN	annualised BTC average emissions (minimum)	kgCO_2_	Authors
BTCEMI_GUE	annualised BTC average emissions (optimal)	kgCO_2_	Authors
BTCOAL_MAX	annualised BTC emissions from coal (maximum)	kgCO_2_	Authors
BTCOAL_MIN	annualised BTC emissions from coal (minimum)	kgCO_2_	Authors
BTCOAL_GUE	annualised BTC emissions from coal (optimal)	kgCO_2_	Authors
BTCOIL_MAX	annualised BTC emissions from oil (maximum)	kgCO_2_	Authors
BTCOIL_MIN	annualised BTC emissions from oil (minimum)	kgCO_2_	Authors
BTCOIL_GUE	annualised BTC emissions from oil (optimal)	kgCO_2_	Authors
BTCGAS_MAX	annualised BTC emissions from gas (maximum)	kgCO_2_	Authors
BTCGAS_MIN	annualised BTC emissions from gas (minimum)	kgCO_2_	Authors
BTCGAS_GUE	annualised BTC emissions from gas (optimal)	kgCO_2_	Authors

Notes: The raw data were converted from the original measurements in TWh to kWh before constructing the emission dataset using IEA emission factors.

**Fig. 1:**
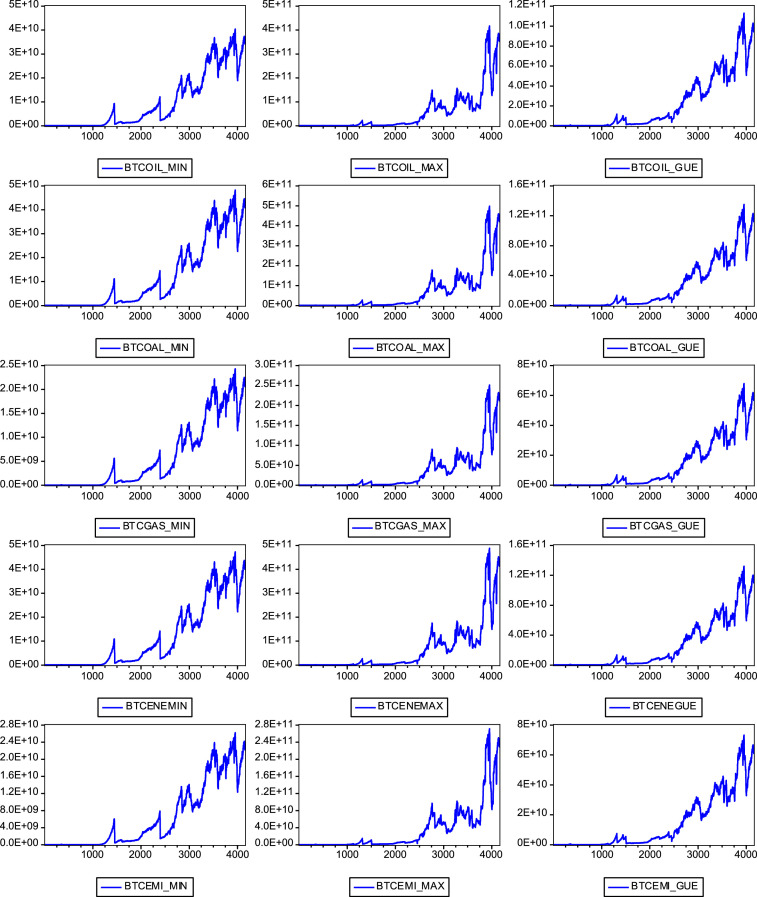
The trend of data on bitcoin energy consumption and carbon footprint.

Fig. 1 depicts the trend of annualised bitcoin energy consumption and carbon footprint for 4,158 data points from July 7, 2010 to December 4, 2021.

Table 2 presents the descriptive statistical analysis of data variables showing the mean, median, maximum, minimum, standard deviation, skewness, kurtosis, and Jarque-Bera test.

**Table 2:** Descriptive statistics of the dataset.

Statistics	Mean	Median	Maximum	Minimum	Std. Dev.	Skewness	Kurtosis	JB	Obs
BTCOIL_MIN	9.71 × 10^9^	3.78 × 10^9^	4.04 × 10^10^	117364.5	1.17 × 10^10^	0.988	2.528	714.582*	4158
BTCOIL_MAX	5.29 × 10^10^	8.75 × 10^9^	4.17 × 10^11^	453766	8.43 × 10^10^	2.302	8.346	8624.297*	4158
BTCOIL_GUE	2.18 × 10^10^	6.81 × 10^9^	1.13 × 10^11^	415952.2	2.81 × 10^10^	1.273	3.592	1183.133*	4158
BTCOAL_MIN	1.16 × 10^10^	4.51 × 10^9^	4.82 × 10^10^	140040.3	1.39 × 10^10^	0.988	2.529	714.568*	4158
BTCOAL_MAX	6.31 × 10^10^	1.05 × 10^10^	4.98 × 10^11^	541437.4	1.01 × 10^11^	2.302	8.344	8620.225*	4158
BTCOAL_GUE	2.60 × 10^10^	8.13 × 10^9^	1.35 × 10^11^	496317.7	3.35 × 10^10^	1.273	3.594	1184.368*	4158
BTCGAS_MIN	5.85 × 10^9^	2.28 × 10^9^	2.43 × 10^10^	70638.59	7.03 × 10^9^	0.988	2.528	714.584*	4158
BTCGAS_MAX	3.18 × 10^10^	5.27 × 10^9^	2.51 × 10^11^	273109.8	5.08 × 10^10^	2.302	8.345	8622.382*	4158
BTCGAS_GUE	1.31 × 10^10^	4.10 × 10^9^	6.79 × 10^10^	250350.6	1.69 × 10^10^	1.272	3.592	1182.559*	4158
BTCENEMIN	1.14 × 10^10^	4.43 × 10^9^	4.73 × 10^10^	137429.2	1.37 × 10^10^	0.988	2.529	714.624*	4158
BTCENEMAX	6.19 × 10^10^	1.03 × 10^10^	4.88 × 10^11^	531341.9	9.87 × 10^10^	2.302	8.345	8622.476*	4158
BTCENEGUE	2.55 × 10^10^	7.98 × 10^9^	1.32 × 10^11^	487063.4	3.29 × 10^10^	1.273	3.593	1183.248*	4158
BTCEMI_MIN	6.30 × 10^9^	2.45 × 10^9^	2.62 × 10^10^	76135.76	7.58 × 10^9^	0.988	2.529	714.613*	4158
BTCEMI_MAX	3.43 × 10^10^	5.68 × 10^9^	2.71 × 10^11^	294363.4	5.47 × 10^10^	2.302	8.347	8626.348*	4158
BTCEMI_GUE	1.41 × 10^10^	4.42 × 10^9^	7.32 × 10^10^	269833.2	1.82 × 10^10^	1.273	3.592	1182.991*	4158

*Notes:* * denotes the rejection of the null hypothesis of normal distribution. JB is the Jarque-Bera test for assessing normal distribution.

**Fig. 2:**
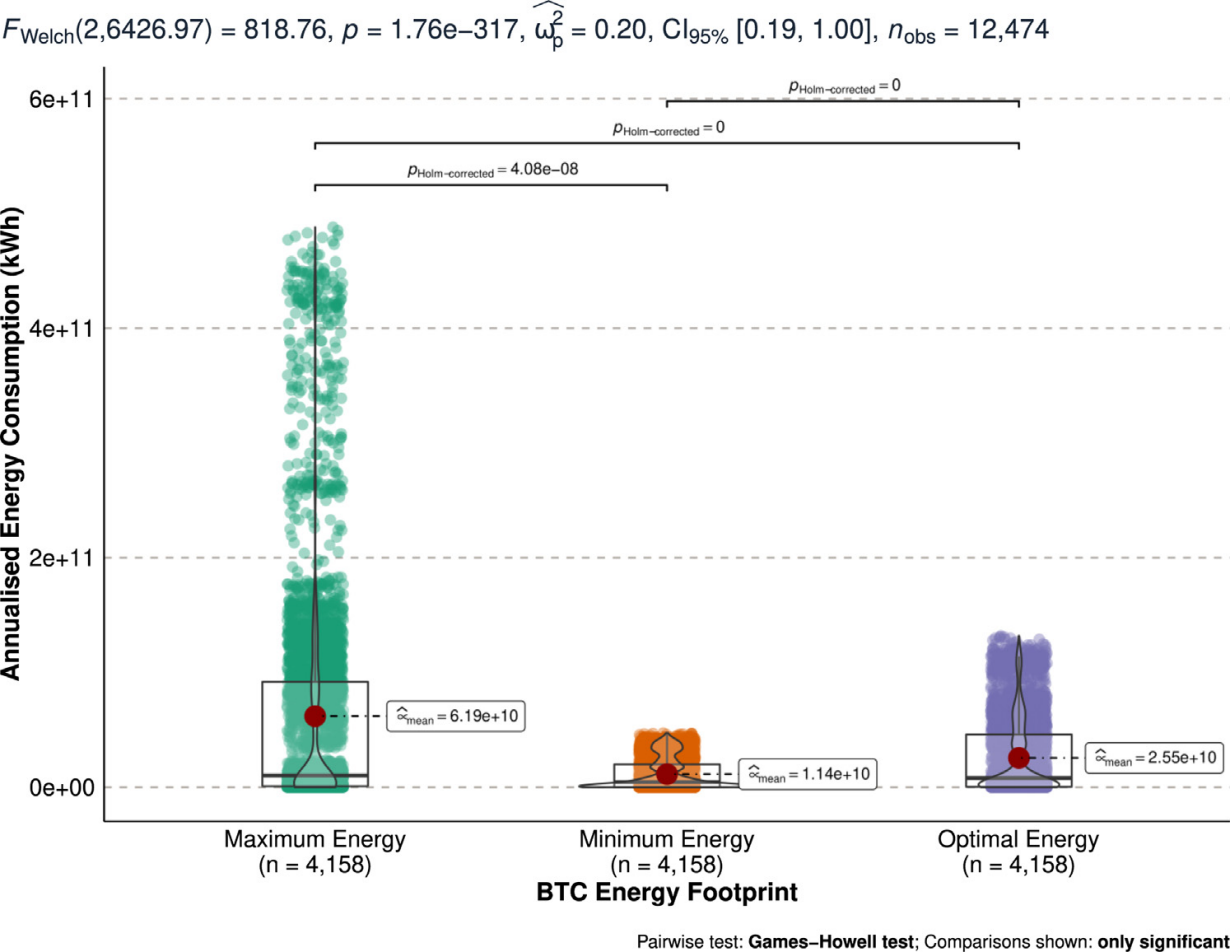
Differences in annualised bitcoin energy consumption (kWh).

Fig. 2 shows the annualised bitcoin energy consumption measured in kWh. Using Fig. 2, the maximum, minimum, and optimal energy consumption of the bitcoin network is compared using the Games-Howell test.

**Fig. 3:**
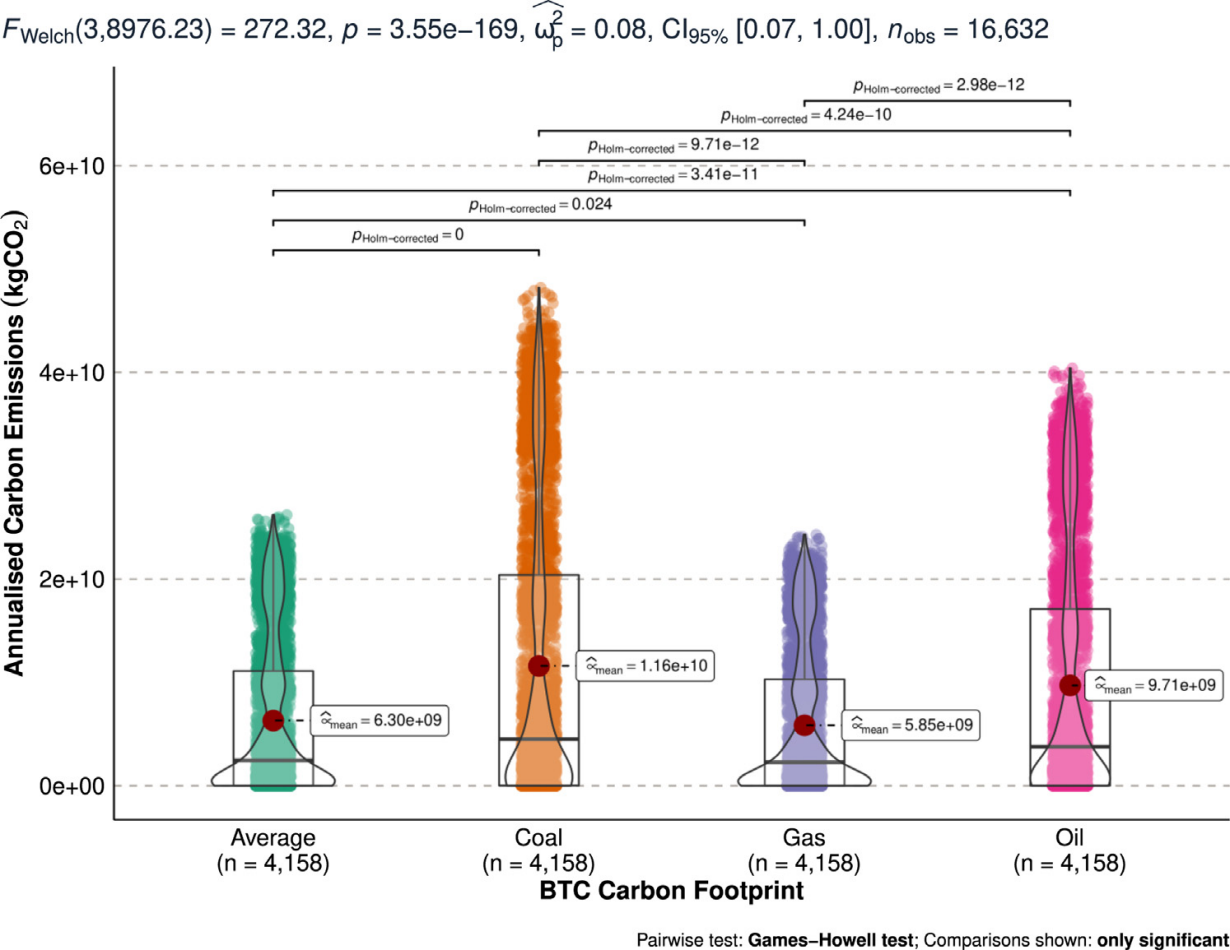
Differences in estimated annualised minimum bitcoin carbon emissions (kgCO_2_).

Fig. 3 shows the annualised minimum bitcoin carbon emissions measured in kgCO_2_. Fig. 3 compares the distribution of the constructed minimum carbon footprint from coal, oil, gas, and average of the 3 energy sources.

**Fig. 4:**
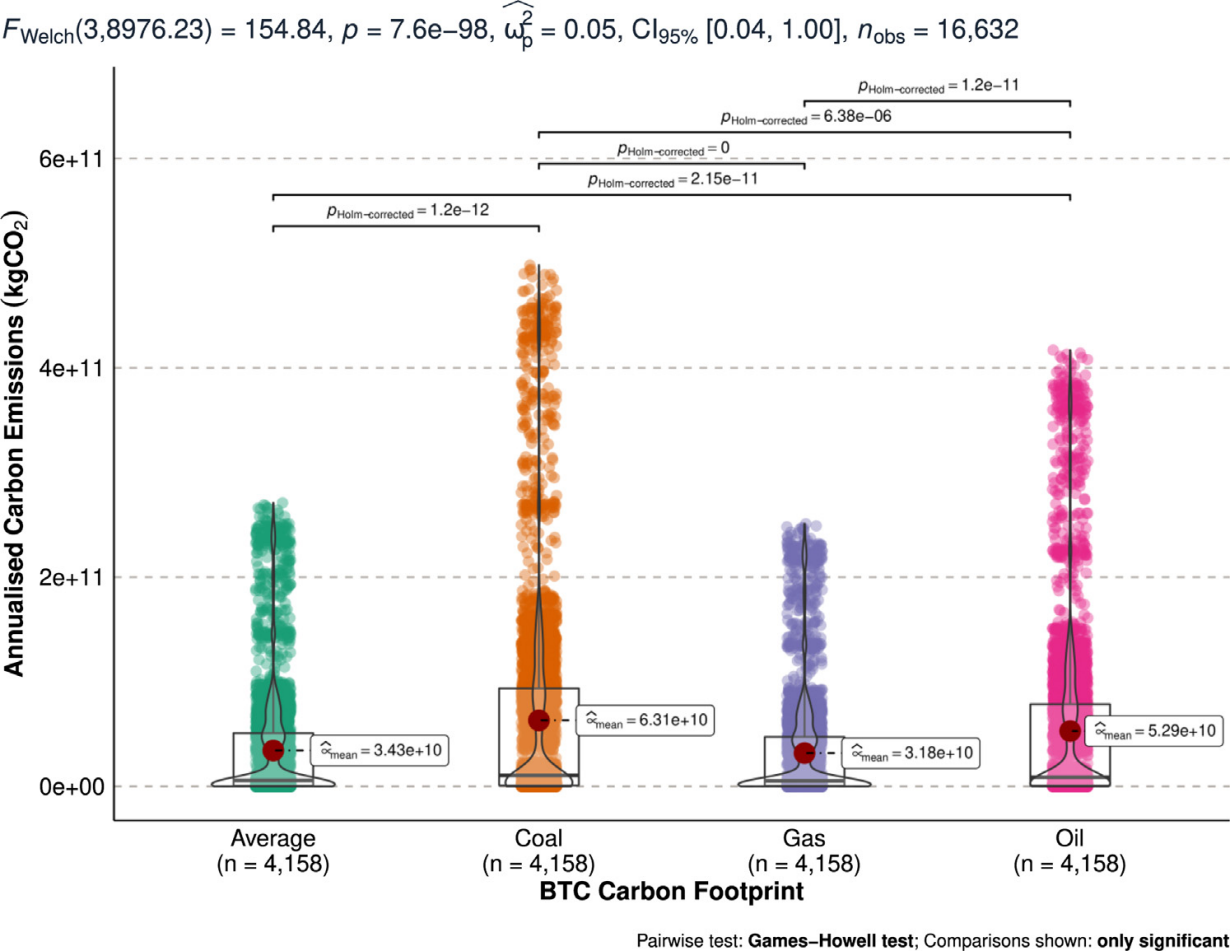
Differences in estimated annualised maximum bitcoin carbon emissions (kgCO_2_).

Fig. 4 shows the annualised maximum bitcoin carbon emissions measured in kgCO_2_. Fig. 4 compares the distribution of the constructed maximum carbon footprint from coal, oil, gas, and average of the 3 energy sources.

**Fig. 5:**
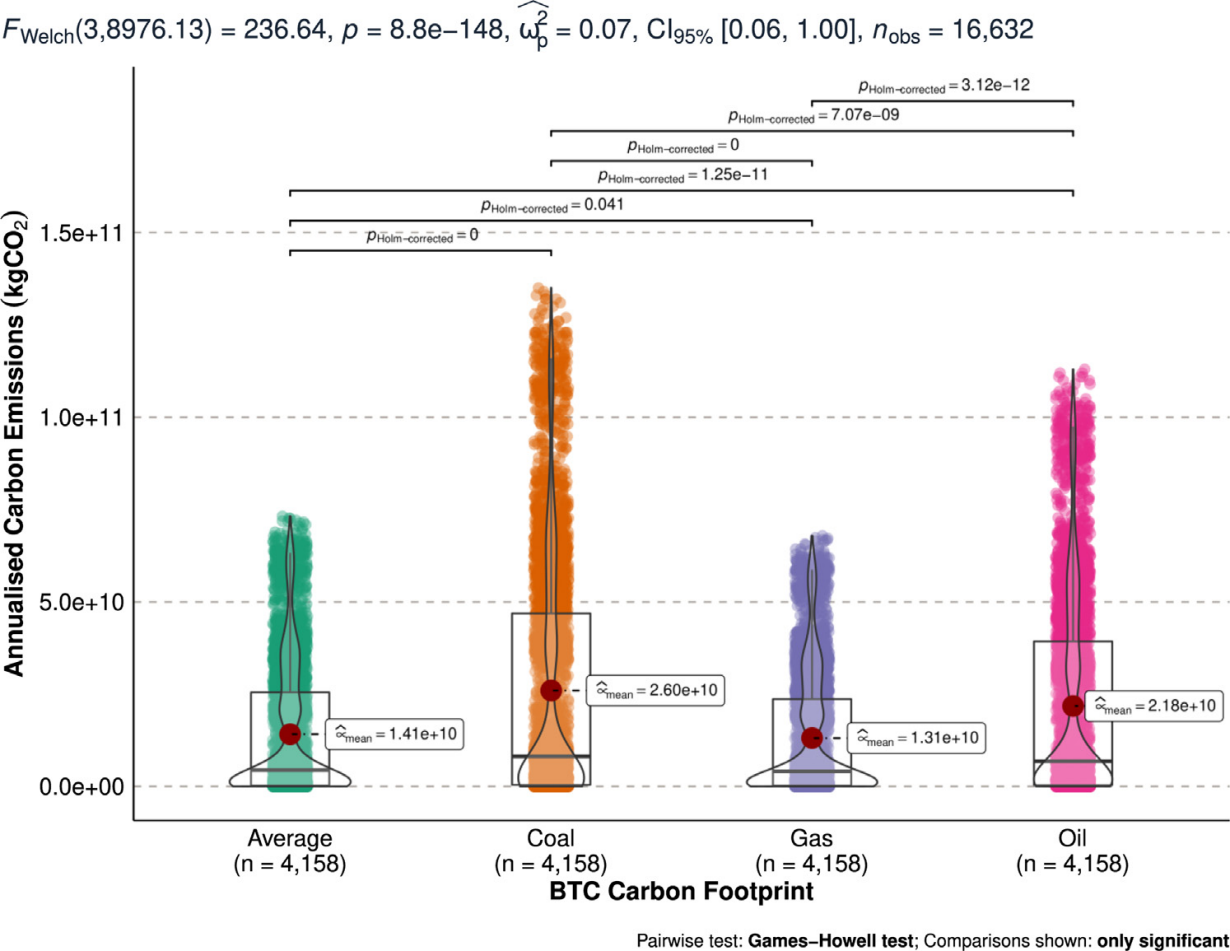
Differences in estimated annualised optimal bitcoin carbon emissions (kgCO_2_).

Fig. 5 shows the annualised optimal bitcoin carbon emissions measured in kgCO_2_. Fig. 5 compares the distribution of the constructed optimal carbon footprint from coal, oil, gas, and average of the 3 energy sources.

**Fig. 6:**
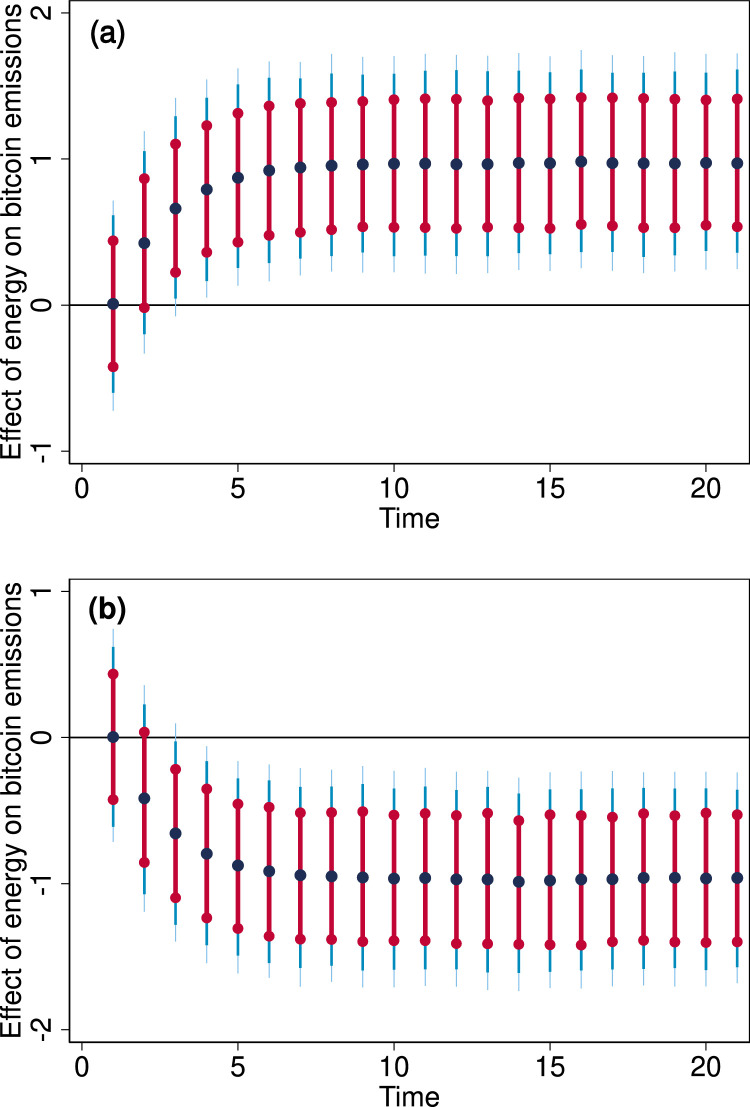
Change in bitcoin carbon footprint for (a) 1% change in bitcoin energy consumption (b) -1% counterfactual shock in bitcoin energy consumption. Notes: (•) is the predicted change. Cranberry, light-blue colours denote the confidence intervals.

Fig. 6 presents the effect of counterfactual change in energy consumption on carbon emissions in the bitcoin network. The change in bitcoin carbon footprint was estimated using the dynamic ARDL simulations––an empirical procedure expounded in Sarkodie and Owusu [2] to examine the relationship between energy consumption and carbon footprint based on the bitcoin network.

## 2 Experimental Design, Materials and Methods

Following the estimation procedure presented in Stoll, Klaaßen and Gallersdörfer [3], the bitcoin carbon footprint [kgCO_2_] CF is calculated as:

(1) CF=EC×EF

Where EC denotes energy consumption [kWh] and EF represents emission factor [kgCO_2_/kWh] that captures carbon intensity of the energy mix namely coal, oil, and gas. Thus, Eq. (1) underpins the daily frequency data on bitcoin carbon footprint constructed using STATA (version 16) and R (version 4.1.2) software. The raw data from CBECI [1] were converted from the original measurements in TWh to kWh before developing the emission dataset using emission factors from IEA World Energy Outlook 2017 Annex A Tables for Scenario Projections. The global emission factors for coal, oil, gas and average are 1.019, 0.854, 0.514, and 0.554 kgCO_2_/kWh, respectively. Which is nearly closer to emission factors presented in de Vries, et. al, [4]. To construct the bitcoin carbon footprint, the following assumptions corresponding to the outlined emission factors are made:

First, energy used for data centres and mining equipment regardless of hardware type is derived solely from coal. Second, energy used for data centres and mining equipment regardless of hardware type is exclusively from oil. Third, energy used for data centres and electricity for mining equipment regardless of hardware type is derived specially from gas. Fourth, energy used for data centres and electricity for mining equipment regardless of hardware type is derived from all the energy mix.

Based on the four assumptions, each of the 3 scenarios of energy consumption namely minimum, maximum, and optimal power consumption are subsequently multiplied by the four emission factors to develop 12 daily frequency data variables of bitcoin carbon footprint. However, caution should be taken in using the bitcoin emission dataset, as the global emission factors are static, yet, emission factor differs across countries. For example, the emission factor for coal-based electricity for Bitcoin miners is ∼50% higher than the global average [4].

## CRediT Author Statement

**Samuel Asumadu Sarkodie:** Conceptualization, Methodology, Software, Visualization, Data curation, Formal analysis, Writing – original draft; **Phebe Asantewaa Owusu:** Writing – original draft, Writing – review & editing.

## Declaration of Competing Interest

The authors declare that they have no known competing financial interests or personal relationships that could have appeared to influence the work reported in this paper.

## Data Availability

Dataset on bitcoin carbon footprint and energy consumption (Original data) (Figshare).

## Appendix Supplementary materials

Image, application 1
